# AMP Is a True Physiological Regulator of AMP-Activated Protein Kinase by Both Allosteric Activation and Enhancing Net Phosphorylation

**DOI:** 10.1016/j.cmet.2013.08.019

**Published:** 2013-10-01

**Authors:** Graeme J. Gowans, Simon A. Hawley, Fiona A. Ross, D. Grahame Hardie

**Affiliations:** 1 Division of Cell Signalling & Immunology, College of Life Sciences, University of Dundee, Dow Street, Dundee DD1 5EH, Scotland, UK

## Abstract

While allosteric activation of AMPK is triggered only by AMP, binding of both ADP and AMP has been reported to promote phosphorylation and inhibit dephosphorylation at Thr172. Because cellular concentrations of ADP and ATP are higher than AMP, it has been proposed that ADP is the physiological signal that promotes phosphorylation and that allosteric activation is not significant in vivo. However, we report that: AMP is 10-fold more potent than ADP in inhibiting Thr172 dephosphorylation; only AMP enhances LKB1-induced Thr172 phosphorylation; and AMP can cause >10-fold allosteric activation even at concentrations 1–2 orders of magnitude lower than ATP. We also provide evidence that allosteric activation by AMP can cause increased phosphorylation of acetyl-CoA carboxylase in intact cells under conditions in which there is no change in Thr172 phosphorylation. Thus, AMP is a true physiological regulator of AMPK, and allosteric regulation is an important component of the overall activation mechanism.

## Highlights

• AMP and ADP both inhibit AMPK dephosphorylation and inactivation, but AMP is more potent
• AMP, but not ADP, promotes Thr172 phosphorylation and AMPK activation, and only by LKB1
• Allosteric activation by AMP is significant even at cellular ATP concentrations
• Allosteric activation of AMPK by AMP occurs in intact cells

## Introduction

AMP-activated protein kinase (AMPK) is a sensor of cellular energy status expressed ubiquitously in almost all eukaryotic cells. Once activated by metabolic stresses that inhibit ATP production or accelerate ATP consumption, it triggers metabolic changes that act to restore energy homeostasis, switching on catabolic pathways that generate ATP while inhibiting anabolic pathways and other ATP-requiring processes (Hardie et al., 2011; 2012). AMPK exists as heterotrimeric complexes comprising catalytic α subunits and regulatory β and γ subunits, each of which occurs in mammals as alternate isoforms encoded by distinct genes. Phosphorylation of Thr172 within the activation loop of the α subunit kinase domain can cause activation of >100-fold in cell-free assays. The major Thr172 kinase is a complex containing the tumor suppressor kinase LKB1 (liver kinase B1) (Hawley et al., 2003; Shaw et al., 2004; Woods et al., 2003), although many cells display an alternate pathway involving the calmodulin-dependent protein kinase, CaMKKβ (Hawley et al., 2005; Hurley et al., 2005; Woods et al., 2005). CaMKKβ is activated by increases in intracellular Ca^2+^, while the LKB1 complex appears to be constitutively active (Lizcano et al., 2004; Sakamoto et al., 2004). However, binding of adenine nucleotides to the γ subunit (Scott et al., 2004; Xiao et al., 2007; 2011) causes conformational changes that regulate the phosphorylation and dephosphorylation of Thr172, and hence AMPK activity, allowing the phosphorylation state to alter according to cellular energy status.

The regulatory adenine nucleotide-binding sites on the γ subunits are formed by four tandem CBS repeats (Scott et al., 2004). Crystallography of partial complexes from mammals and fungi (Amodeo et al., 2007; Jin et al., 2007; Townley and Shapiro, 2007; Xiao et al., 2007; 2011) revealed that these have a pseudosymmetrical layout, generating four clefts in the center where adenine nucleotides could bind; these sites are numbered according to which CBS repeat bears an aspartate side chain involved in nucleotide binding (Kemp et al., 2007). In a structure of a mammalian complex crystallized with AMP, site 2 was empty, while sites 1, 3, and 4 were occupied by AMP. When ATP was soaked into the crystals, AMP was replaced by ATP only at sites 1 and 3, so site 4 was designated a nonexchangeable site where AMP was proposed to be permanently bound (Xiao et al., 2007; 2011). Competitive binding studies using fluorescent ATP derivatives suggested that the affinities for binding of AMP, ADP, and ATP at sites 1 and 3 are similar, although site 1 appeared to have an affinity 30- to 40-fold higher than that of site 3 for all three nucleotides (Xiao et al., 2011).

Even before the identity of the upstream kinases had been determined, AMP binding had been reported to both promote phosphorylation (Hawley et al., 1995) and inhibit dephosphorylation (Davies et al., 1995) of Thr172. It was recently reported that binding of ADP, as well as AMP, inhibited dephosphorylation (Xiao et al., 2011) and that ADP as well as AMP enhanced phosphorylation of Thr172 by both LKB1 and CaMKKβ (Oakhill et al., 2010; 2011). Based on these findings and the fact that cellular ADP concentrations are usually at least one order of magnitude higher than those of AMP, it was proposed that ADP, not AMP, is the physiological signal that enhances net Thr172 phosphorylation and that allosteric activation by AMP may not be relevant in the physiological context (Carling et al., 2012; Oakhill et al., 2012). In this paper, we have reinvestigated these questions.

## Results

### AMP Is More Potent than ADP in Inhibiting Dephosphorylation of Thr172

**Figure 1:**
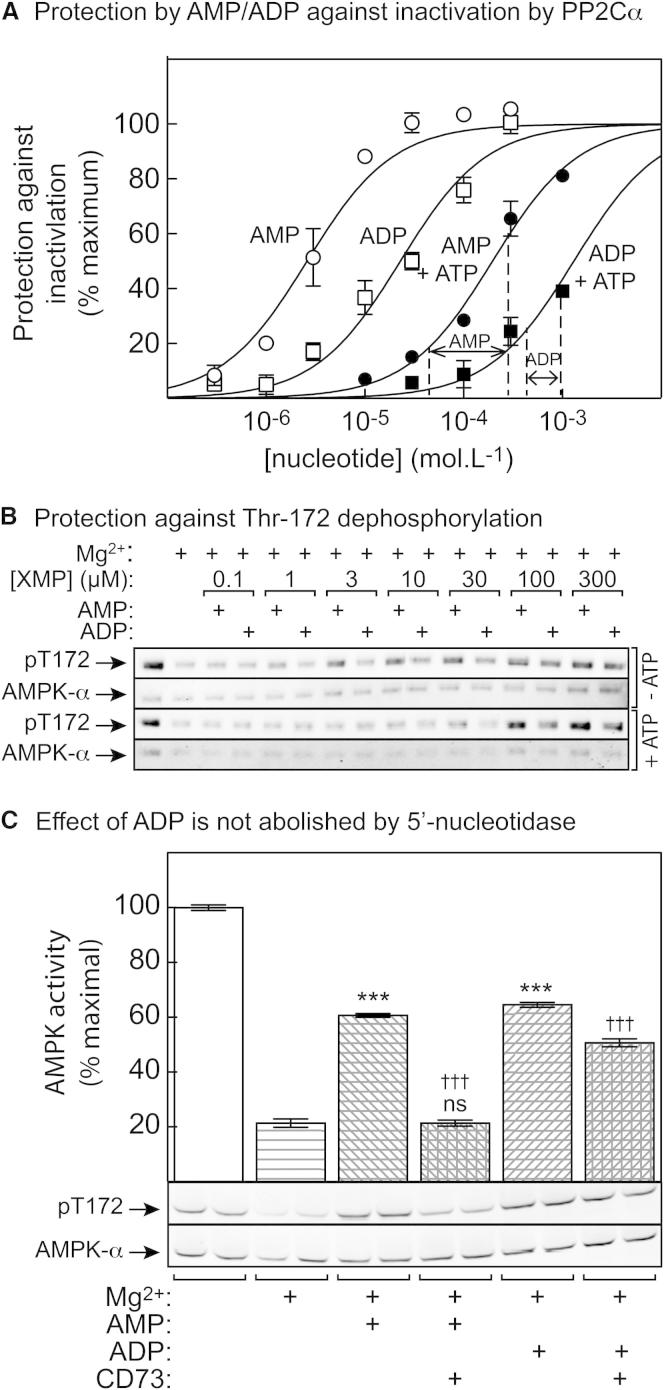
Effect of AMP, ADP, and 5′-Nucleotidase on the Inactivation and Thr172 Dephosphorylation of AMPK by PP2Cα Incubations without Mg^2+^ were used as controls. (A) Effects of AMP and ADP on inactivation by phosphatase (PP2Cα) ± 5 mM ATP. Protection against inactivation is defined as the activity difference in assays with or without nucleotide expressed as a percentage of the difference obtained with or without optimal nucleotide (30 μM for AMP and 300 μM for ADP, both in the absence of ATP). Results were fitted to the equation: Y = 100 × X / (EC_50_ + X), where Y is the percent of protection and X is the concentration of AMP or ADP. Data are mean ± SEM (n = 2), and curves were generated with the equation above, using best-fit values for EC_50_ quoted in the text. The vertical dotted lines show the ranges over which AMP and ADP concentrations were estimated to change when G361 cells were treated with 100 μM berberine (Table S1 and Figure 5). (B) Samples from incubations ± 5 mM ATP were analyzed by western blotting using anti-pT172 and anti-AMPK-α antibodies. (C) AMPK was incubated at 30°C with PP2Cα ± Mg^2+^ as in (A), with or without AMP (100 μM), ADP (300 μM), or the 5′-ectonucleotidase CD73 as indicated. The nucleotides were preincubated with CD73 for 5 min at 30°C before starting the reaction by the addition of AMPK. Kinase activities (expressed as percentages of activity in control without Mg^2+^, mean ± SEM, n = 3) and anti-pT172 blots are shown. ^∗∗∗^Significantly different from controls without AMP/ADP (p < 0.001); ^†††^significantly different from control without CD73 (p < 0.001); ns, not significantly different from control without AMP or CD73.

Native AMPK purified from rat liver has been consistently reported to exhibit a greater allosteric activation by AMP (typically 3- to 4-fold; Carling et al., 1987; 1989) than bacterially expressed rat or human complexes (typically 1.5- to 2-fold; Sanders et al., 2007; Suter et al., 2006). We therefore used purified rat liver AMPK to reinvestigate the regulatory effects of adenine nucleotides in cell-free assays. We first monitored the ability of various concentrations of AMP and ADP to protect against inactivation caused by incubation with recombinant PP2Cα (Figure 1A). This confirmed previous results (Xiao et al., 2011) showing that ADP, as well as AMP, protected against inactivation and dephosphorylation, but AMP was effective at lower concentrations than ADP. In the absence of ATP, the half-maximal effect of AMP on inactivation (EC_50_) was at 2.6 ± 0.3 μM, whereas the half-maximal effect of ADP was at 23 ± 3 μM, almost 10-fold higher. We repeated the assays in the presence of 5 mM ATP (Figure 1A). As expected if ATP competes with AMP and ADP, the EC_50_ for AMP increased by 75-fold to 196 ± 15 μM and for ADP by 60-fold to 1.4 ± 0.1 mM. However, the higher potency of AMP compared with that of ADP was retained. At this ATP concentration (which is within the physiological range), the EC_50_ values for AMP and ADP were 25-fold and 3- to 4-fold lower, respectively, than the ATP concentration. Parallel analysis of Thr172 phosphorylation (Figure 1B) was consistent with the activity assays.

Analysis of our commercial preparation of ADP showed that it exhibited ≈1% contamination with AMP. Thus, contamination with AMP was unlikely to explain an effect of ADP that was only 10-fold less potent than that of AMP. However, to rule this out we repeated the assays at saturating concentrations of AMP and ADP with and without the 5′-nucleotidase CD73, which hydrolyzes AMP to adenosine and phosphate, but not ADP or ATP. While the effects of AMP were abolished as expected, the effects of ADP were only partially reduced (Figure 1C). The small reduction may be accounted for by hydrolysis of the small amount of AMP contaminating the ADP, but the residual protection must be due to ADP.

### AMP, but Not ADP, Enhances Phosphorylation by LKB1, but Not CaMKKβ

**Figure 2:**
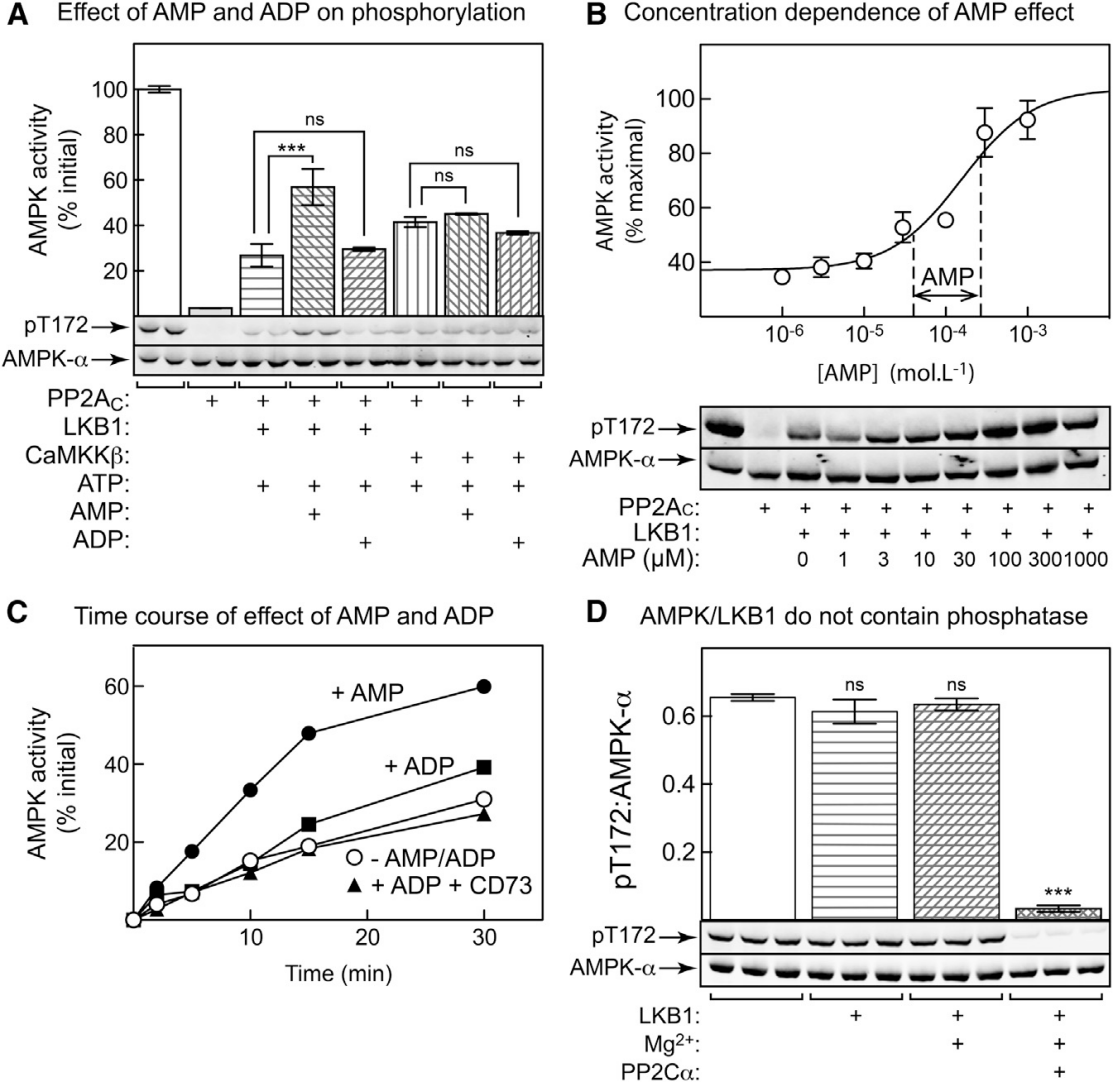
AMP, but Not ADP, Enhances Thr172 Phosphorylation and Activation of AMPK by LKB1, but Not CaMKKβ (A) Effect of AMP and ADP on activation and Thr172 phosphorylation by LKB1 and CaMKKβ. Purified rat liver AMPK was incubated with bacterially expressed human PP2A_C_ to give ≈95% inactivation. Okadaic acid was then added to inhibit PP2A_C_, and the dephosphorylated kinase was incubated for 10 min with MgCl_2_ (5 mM) and ATP (200 μM), with or without LKB1 or CaMKKβ, and with or without AMP or ADP (300 μM). Aliquots were taken for kinase assays (top; mean ± SEM, n = 3) or western blotting (bottom). Kinase activities are expressed as percentages of the initial kinase activity prior to dephosphorylation. (B) Concentration dependence of the effect of AMP on AMPK activation by LKB1 (other conditions are as in A; mean ± SEM, n = 3). Data were fitted to the equation Y = basal + ([{activation × basal − basal} × X] / [EC_50_ + X]), where Y is kinase activity and X is AMP concentration. The curve was generated using the following best-fit parameters: basal, 37% ± 3%; activation, 2.8 ± 0.3-fold; EC_50_, 160 ± 60 μM. The vertical dotted lines show the range over which AMP concentration was estimated to change when G361 cells were treated with 100 μM berberine (Table S1 and Figure 5). (C) Time course of AMPK activation by LKB1. Incubations contained no additions (open circles), 300 μM AMP (filled circles), 300 μM ADP (squares), or 300 μM ADP plus the 5′-nucleotidase CD73 (triangles). Kinase activities are expressed as percentages of the initial kinase activity prior to dephosphorylation. (D) Rat liver AMPK was incubated with or without LKB1 (amounts as in A), with or without MgCl_2_ (5 mM), and with or without PP2Cα, and samples were analyzed by western blotting. The bar chart at the top (mean ± SEM, n = 3) shows quantification by densitometry of the blots at the bottom (ratio of signal with anti-pT172 and anti-AMPK-α, arbitrary units). ^∗∗∗^Significantly different (p < 0.001); ns, not significantly different from control without additions by one-way ANOVA with Dunnett’s multiple comparison test.

Both AMP and ADP have recently been reported to enhance phosphorylation of Thr172 by LKB1 and CaMKKβ, as long as the β subunit was N-myristoylated (Oakhill et al., 2010; 2011). Figure 2A shows that AMP promoted activation and Thr172 phosphorylation of rat liver AMPK by LKB1, but not CaMKKβ. We were unable to discern a significant effect of the same concentration of ADP (300 μM) with either upstream kinase. Figure 2B shows the dependence of activation by LKB1 on AMP concentration; it caused 2.8 ± 0.2-fold stimulation with EC_50_ at 160 ± 60 μM AMP. This relatively high EC_50_ would explain why contamination of 300 μM ADP with 1% AMP would not be sufficient to generate an apparent effect of ADP. Figure 2C show time courses for the effects of AMP and ADP on activation of AMPK by LKB1. At the 10 min time point (as used in Figure 2A), we did not observe any effect of ADP, although a small stimulatory effect became evident at later time points. This appeared to be due to generation of AMP from ADP during the assay, because it was abolished by the inclusion of 5′-nucleotidase (Figure 2C).

Our original demonstration that AMP promoted Thr172 phosphorylation on rat liver AMPK (Hawley et al., 1995) by upstream kinase (later shown to be an LKB1-STRADα-MO25α complex; Hawley et al., 2003) was questioned on the basis that the effects might be explained by contamination with a Mg^2+^-dependent, protein phosphatase M (PPM) family phosphatase. Inhibition by AMP of Thr172 dephosphorylation by a PPM phosphatase, which would be insensitive to the added okadaic acid, would lead to an apparent increase in net phosphorylation (Sanders et al., 2007). Our AMPK preparation is purified ≈1,000-fold through six purification steps, whereas that used by Sanders et al. (2007) was only purified through the first two. Nevertheless, to rule this out we carried out mock phosphorylation incubations lacking ATP, which showed that incubation of AMPK with LKB1 and Mg^2+^ caused no Thr172 dephosphorylation, unless exogenous PP2Cα was added (Figure 2D).

### AMP Causes a Large Allosteric Activation of AMPK, Even at High ATP Concentrations

**Figure 3:**
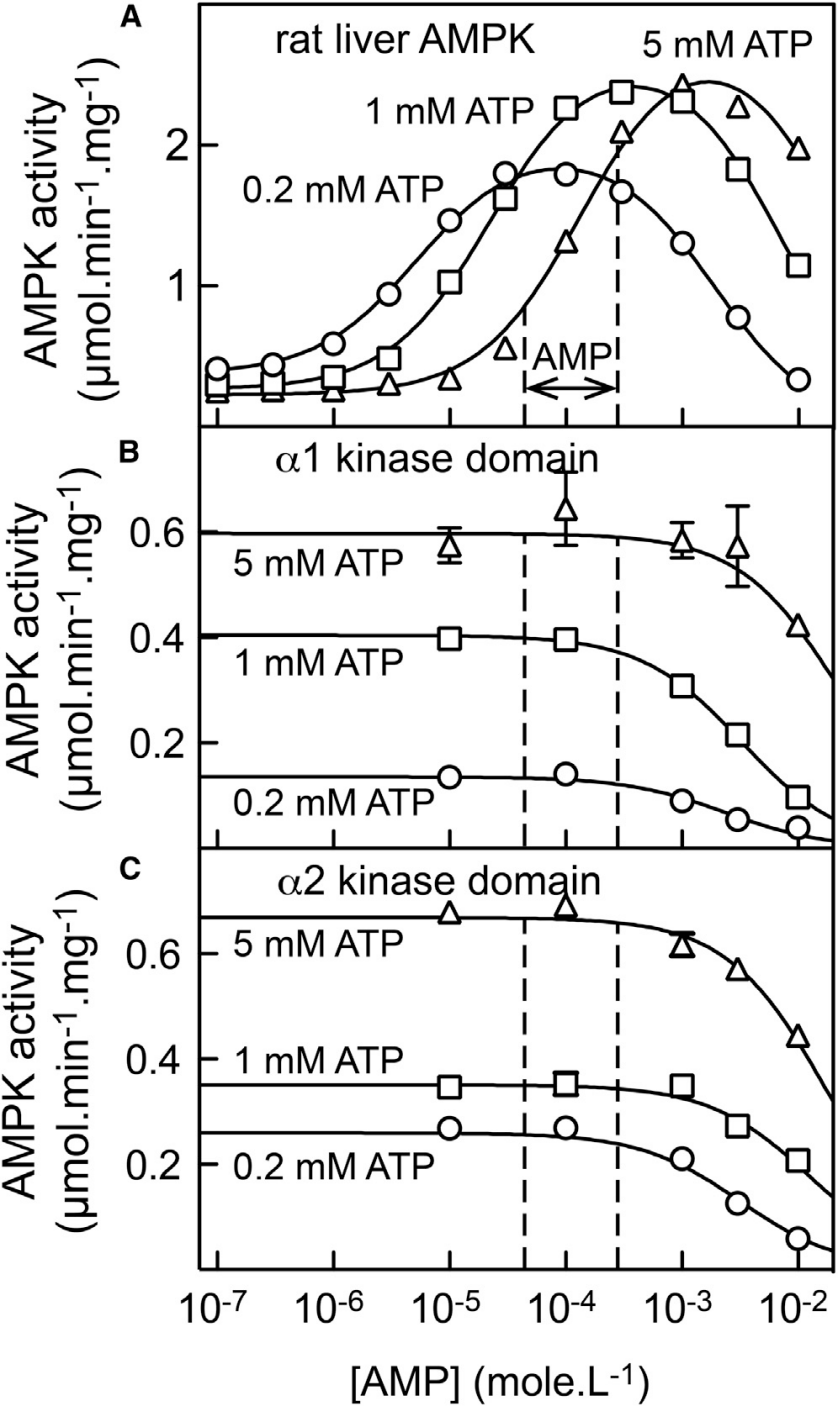
Allosteric Activation by AMP, and Inhibition at High AMP Concentrations, of Rat Liver AMPK and GST Fusions of the Isolated α1 and α2 Kinase Domains (A) Effect of increasing AMP concentrations on the activity of purified rat liver AMPK. Data points were generated at three different concentrations of ATP (circles, 0.2 mM; squares, 1 mM; triangles, 5 mM) and were fitted to the equation Y = basal + ([{activation × basal − basal} × X] / [EC_50_ + X]) − ([{activation × basal} × X] / [IC_50_ + X]), where Y is activity and X is the AMP concentration. The continuous curves shown were derived using this equation and the best-fit parameters quoted in the main text. (B and C) Effect of increasing AMP concentrations on the activity of GST fusions with the kinase domains of human α1 (B) and α2 (C). Data (mean ± SEM, n = 3) were obtained at three different concentrations of ATP as in (A) and were fitted to the equation Y = basal − (basal × X / [IC_50_ + X]). The continuous curves shown were derived using this equation and the best-fit parameters obtained (±SEM at 0.2, 1, and 5 mM ATP, respectively): α1 basal activity, 136 ± 5, 404 ± 6, and 598 ± 22 nmol/min/mg; IC_50_ for α1, 2.4 ± 0.4, 3.3 ± 0.3, and 23 ± 68 mM; α2 basal activity, 260 ± 6, 351 ± 7, and 669 ± 8 nmol/min/mg; IC_50_ for α2, 3.2 ± 0.4, 13 ± 1.4, and 18 ± 1.4 mM. The vertical dotted lines show the range over which AMP concentration was estimated to change when G361 cells were treated with 100 μM berberine (Table S1 and Figure 5).

Using a high-pressure liquid chromatography (HPLC)-based assay that allowed simultaneous measurement of peptide substrate and product and adenine nucleotides, it was reported that commercial preparations of ATP were contaminated with low levels of AMP, which could also be generated during assays, most likely by nonenzymic breakdown of ADP (Suter et al., 2006). To minimize these problems, we utilized freshly prepared ATP and very short assay times (5 min). To address the question of whether AMP is capable of competing with ATP at the allosteric sites, we also conducted assays not only at 200 μM ATP as in the standard assay, but also at the more physiological ATP concentrations of 1 and 5 mM. We obtained a family of bell-shaped curves in which AMP activated AMPK at low concentrations and then inhibited at higher concentrations (Figure 3A). The inhibitory effects were due to competition of AMP with ATP at the catalytic site, because if we assayed bacterially expressed glutathione S-transferase (GST) fusions of the isolated α1 or α2 kinase domains (after phosphorylation by LKB1), we no longer observed activation by AMP but still observed the inhibitory effects (Figures 3B and 3C); these only occurred at AMP concentrations above 1 mM, which are unlikely to be physiologically relevant.

The bell-shaped curves in Figure 3A shifted to higher AMP concentrations as ATP increased, consistent with the idea that ATP competes with AMP both at the activating site(s) on the γ subunit and at the catalytic site on the α subunit. When we fitted the data to a simple model (see Figure 3 legend) that assumed single activating and inhibitory sites for AMP, we obtained good fits (continuous curves in Figure 3A) with the following parameters (±SEM at 0.2, 1, and 5 mM ATP, respectively): basal activity = 372 ± 16, 266 ± 14, and 226 ± 23 μmol/min/mg; EC_50_ for activation by AMP = 5.3 ± 0.4, 22 ± 1.1, and 137 ± 14 μM; IC_50_ for inhibition by AMP = 1.9 ± 0.092, 7.1 ± 0.33, and 22 ± 3 mM; degree of activation by AMP = 5.4 ± 0.2, 10 ± 0.5, and 13 ± 1.3-fold. Thus, even when the ATP concentration was 5 mM (similar to the concentration estimated in unstressed cells; Imamura et al., 2009), AMP caused a large allosteric activation (13-fold) with a half-maximal effect at 140 μM.

### Increased Phosphorylation of Acetyl-CoA Carboxylase in LKB1 Null Cells Treated with AMPK Activators

**Figure 4:**
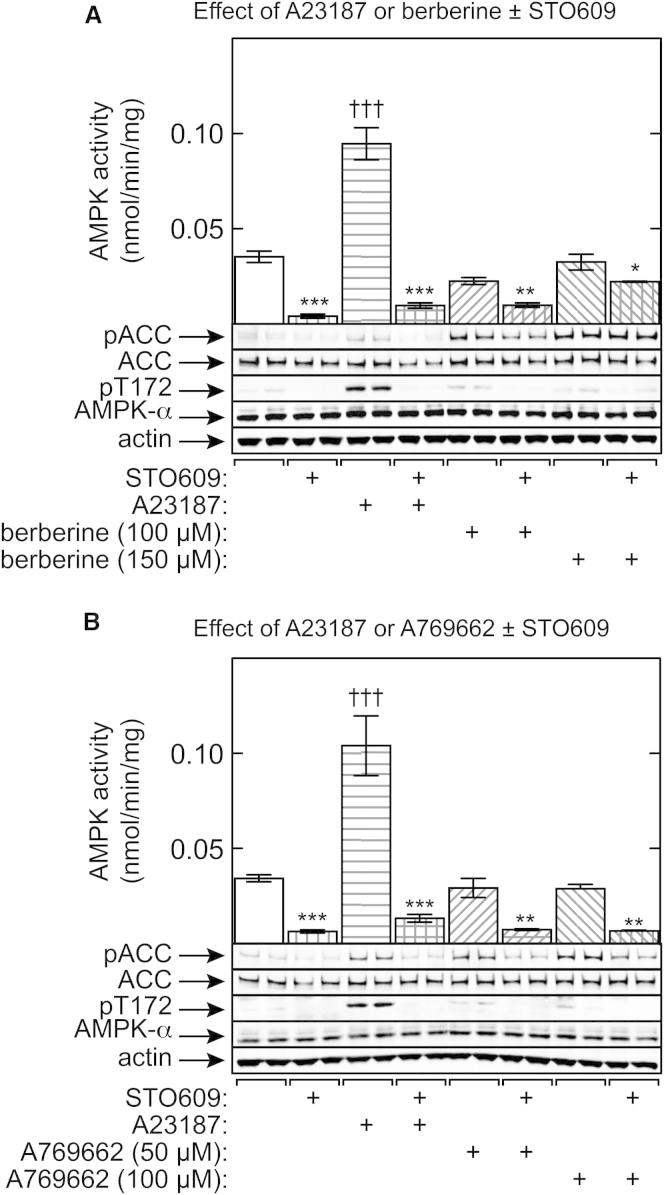
Effects of AMPK Activators and the CaMKK Inhibitor STO609 on Phosphorylation and Activation of AMPK and Phosphorylation of ACC in G361 Cells (A) Effects of A23187 (10 μM) or berberine (100 and 150 μM) in the presence and absence of STO609 (2.5 μM). (B) Effects of A23187 (10 μM) or A769662 (50 and 100 μM) in the presence and absence of STO609 (2.5 μM). Cells were incubated with the indicated agents for 1 hr, and lysates were analyzed using immunoprecipitate kinase assays (mean ± SD, n = 3) and western blotting. Significantly different from control without STO609: ^∗^p < 0.05, ^∗∗^p < 0.01, ^∗∗∗^p < 0.001; significantly different from control without AMPK activator: ††† p < 0.001.

To study the relative importance of allosteric activation versus Thr172 phosphorylation in intact cells, we initially utilized G361 cells, a human melanoma line that lacks LKB1. In LKB1 null cells, agents that increase cellular AMP do not increase Thr172 phosphorylation (Hawley et al., 2003), so they should work entirely via allosteric mechanisms, while agents that increase intracellular Ca^2+^ and activate CaMKKβ work entirely via increased Thr172 phosphorylation. As expected, in G361 cells the natural product berberine (an inhibitor of respiratory chain complex I; Hawley et al., 2010) did not cause increased phosphorylation of Thr172 and failed to increase AMPK activity measured in washed immunoprecipitates (in which any effects of allosteric activation in the intact cell would have been lost). By contrast, the Ca^2+^ ionophore A23187 markedly increased Thr172 phosphorylation and kinase activity (Figure 4A). Despite this, berberine increased phosphorylation of the downstream target acetyl-CoA carboxylase (ACC) to a larger extent than A23187 did. As expected, the CaMKK inhibitor STO609 blocked the effect of A23187 on AMPK activation and Thr172 phosphorylation. It also reduced the low Thr172 phosphorylation and AMPK activities observed under basal conditions and after berberine and reduced the effect of 100 μM berberine on ACC phosphorylation, suggesting that the low, basal CaMKKβ activity was sufficient to generate some Thr172 phosphorylation even without A23187 treatment.

We carried out similar experiments using another AMPK activator, A769662, which does not increase cellular AMP or ADP but acts instead by direct binding to AMPK at site(s) distinct from those used by adenine nucleotides, causing both allosteric activation and inhibition of Thr172 dephosphorylation (Göransson et al., 2007; Hawley et al., 2010; 2012). The effects of A23187 and two different concentrations of A769662 on ACC phosphorylation were very similar and were reduced by STO609 (Figure 4B). However, the results with A769662 bore similarities to those with berberine in that A769662 had no effect on AMPK activity or Thr172 phosphorylation, despite the fact that it strongly enhanced ACC phosphorylation. Thus, like berberine, in these cells A769662 appears to be promoting ACC phosphorylation entirely by allosteric activation of AMPK.

**Figure 5:**
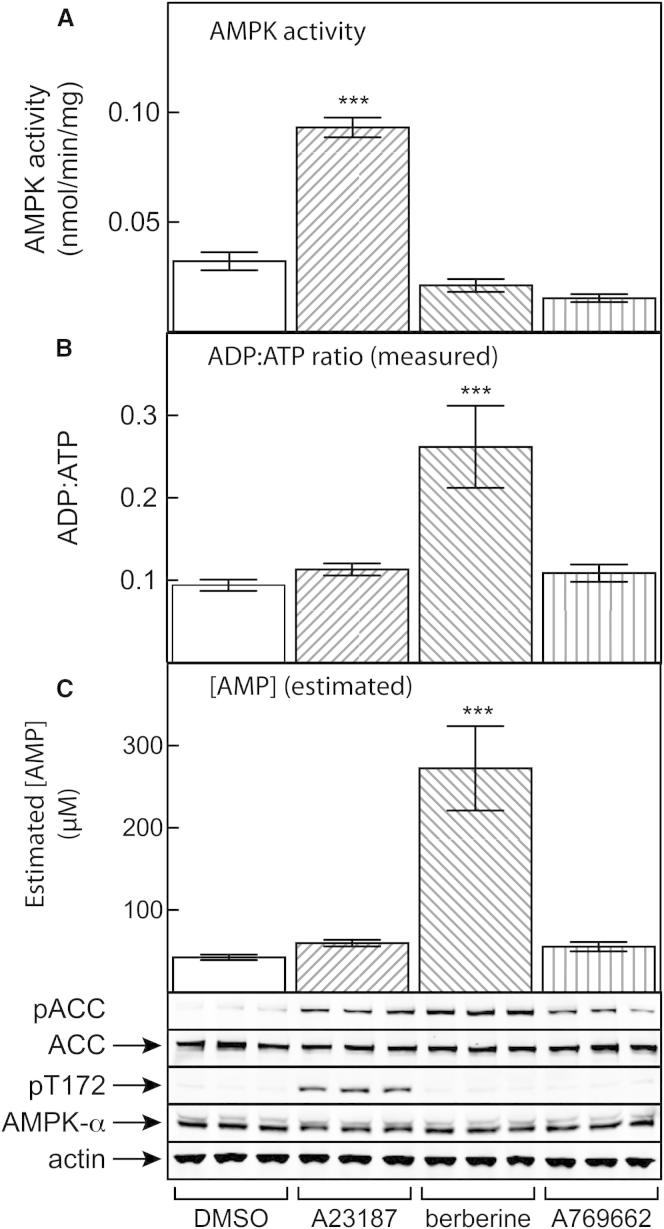
Effects of Treatment of G361 Cells with AMPK Activators on Phosphorylation of ACC, Phosphorylation and Activity of AMPK, Cellular ADP:ATP Ratios, and Calculated Cellular AMP (A–C) Effects of treatment of G361 cells with AMPK activators on phosphorylation of ACC, phosphorylation and activity of AMPK (A), cellular ADP:ATP ratios (B), and calculated cellular [AMP] (C). Replicate dishes of cells (n = 3) were incubated for 1 hr with A23187 (10 μM), berberine (100 μM), A769662 (100 μM), or an equivalent concentration of vehicle (DMSO) and then lysed in parallel in neutral detergent buffer for measurement of AMPK activity in immunoprecipitates (top) or western blotting (bottom), or in perchloric acid for analysis of ADP:ATP ratios (center). AMP concentrations (see Table S1) were calculated as described in Experimental Procedures. Data are mean ± SD. Significantly different from DMSO control: ^∗∗∗^p < 0.001.

Figure 5 shows the results of experiments in G361 cells in which the effects of A23187, berberine, and A769662 on AMPK activity, Thr172 phosphorylation, and cellular ADP:ATP ratios were compared. All three agents caused increased ACC phosphorylation, but only A23187 caused increased Thr172 phosphorylation or AMPK activity. As expected, berberine increased the cellular ADP:ATP ratio, unlike A23187 or A769662 (Figure 5B). The cellular AMP content was too low to directly measure using our methodology, but we estimated its cellular concentration from the ADP:ATP ratios (Table S1, available online, and Figure 5C) by assuming that the adenylate kinase reaction was at equilibrium. Estimated AMP concentrations did not change in response to A23187 or A769662 but increased 6- to 7-fold in response to berberine (from 42 ± 3 to 270 ± 50 μM). This corresponds well with the range of concentrations over which we found that AMP causes inhibition of dephosphorylation (see Figure 1A, where the estimated change in ADP is also shown) and allosteric activation (see Figure 3A) when these effects were measured in cell-free assays in the presence of 5 mM ATP. It also corresponds with the concentrations at which AMP promoted phosphorylation (see Figure 2B), although that was only measured at 200 μM ATP.

### ACC Phosphorylation Is Mediated by AMPK

**Figure 6:**
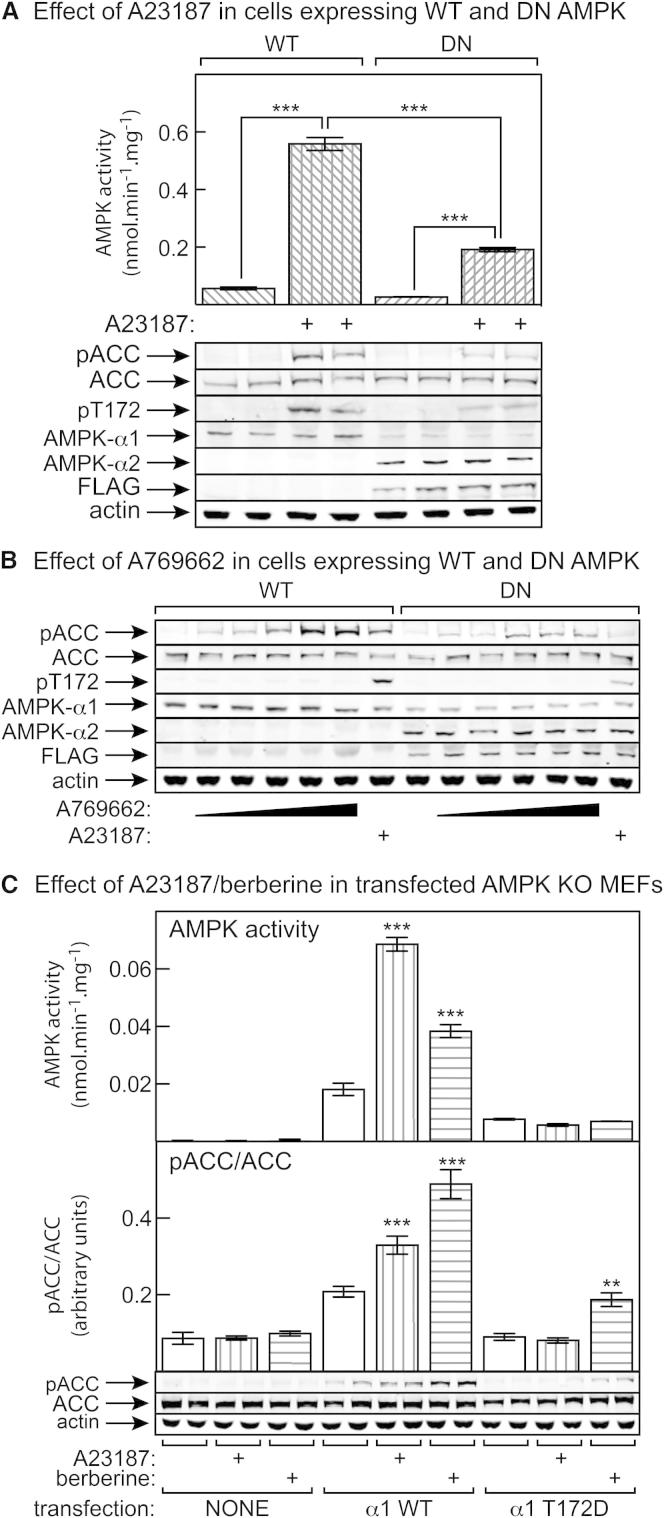
Evidence that ACC Phosphorylation in G361 Cells Is Mediated by AMPK, and Effects of A23187 and Berberine in AMPK Knockout MEFs Expressing α1-T172D Mutant (A) FLAG-tagged inactive (D157A) mutant of AMPK-α2 was stably expressed by homologous recombination in G361 cells carrying an Flp recombinase target site (see Experimental Procedures) to generate dominant-negative (DN) cells. The graph shows AMPK activity (mean ± SEM, n = 4) measured in immunoprecipitates from control (WT) and DN cells with and without treatment with 10 μM A23187, while the pictures below show results of western blotting to determine expression and phosphorylation of various proteins. (B) As in (A), except the cells were treated with increasing concentrations of A769662 (30, 100, 300, 500, 1000 μM) or 10 μM A23187. (C) Effects of A23187 and berberine on AMPK activity and ACC phosphorylation in AMPK KO MEFs that were either untransfected or had been transfected with DNAs encoding myc-tagged AMPK-α1 (wild-type or T172D mutant), AMPK-β2, and AMPK-γ1. Data are mean ± SEM (n = 4). Significant differences from control without A23187 or berberine are shown.

To confirm that ACC phosphorylation in G361 cells was catalyzed by AMPK, we generated cells stably expressing a FLAG-tagged, kinase-inactive mutant (D157A) of AMPK-α2. This mutant was expected to act in a dominant-negative manner by competing with the endogenous α subunits (primarily α1) for binding to β and γ. Figure 6A confirms that these cells did express recombinant α2 as shown by probing blots with anti-α2 or anti-FLAG antibodies; the expression of α1 was reduced, but not eliminated. In addition, basal and A23187-stimulated AMPK activity was decreased by ≈70% (Figure 6A), confirming that the mutant was exerting a dominant-negative effect. The phosphorylation of ACC in response to both A23187 and A769662 was reduced to an extent similar to that of the reduction in AMPK activation (Figure 6), confirming that both effects were mediated largely, if not entirely, by AMPK.

### Berberine Increases ACC Phosphorylation, Even in Cells Expressing a T172D Mutant

As another approach to assess the role of allosteric activation, we used AMPK-α1^−/−^ and AMPK-α2^−/−^ mouse embryo fibroblasts (AMPK KO MEFs) (Laderoute et al., 2006) and coexpressed either wild-type myc-tagged AMPK-α1 or a T172D mutant with AMPK-β2 and AMPK-γ1. The T172D mutant cannot be phosphorylated and activated by upstream kinases but has significant activity and retains allosteric activation by AMP (Stein et al., 2000). We could not detect the expressed proteins using anti-myc, anti-α1, or anti-pT172 antibodies, probably due to low transfection efficiency. Despite this, we could detect a low, but significant, basal AMPK activity in the cells expressing the T172D mutant and a higher basal activity in the cells expressing the wild-type (Figure 6C). As expected, the wild-type activity increased following treatment with A23187 or berberine, but that of the T172D mutant did not. Despite this, phosphorylation of ACC increased significantly in response to berberine in the cells expressing the mutant, which must have been due to allosteric activation. Also consistent with this, the effect of berberine on ACC phosphorylation in the cells expressing wild-type α1 was greater than that of A23187, despite the fact that the effect on AMPK activation was smaller.

### Increases in Thr172 Phosphorylation in Intact Cells Are Modest in Extent

Although bacterially expressed AMPK complexes can be activated >100-fold in cell-free assays by stoichiometric phosphorylation of Thr172 (Suter et al., 2006), it remained unclear whether the degree of Thr172 phosphorylation is ever close to this maximal extent in intact cells. To address this question, we further analyzed the effects of the Ca^2+^ ionophore A23187 in G361 cells and A23187 and berberine in human embryonic kidney 293 (HEK293) cells and estimated, using two different methods, the proportion of the α subunits that became phosphorylated.

**Figure 7:**
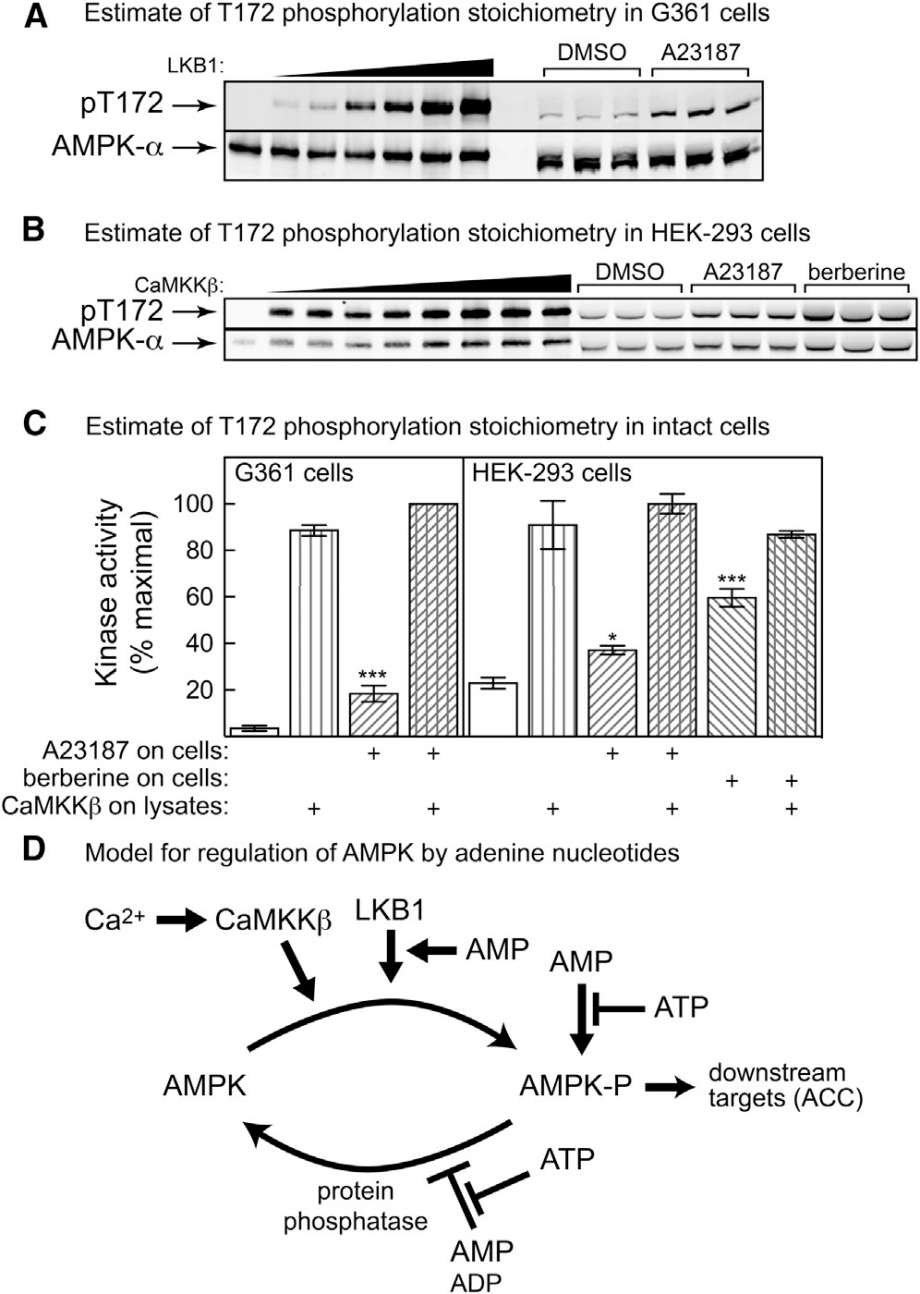
Estimation of Extent of Thr172 Phosphorylation in Intact Cells, and Model for Regulation of AMPK by Adenine Nucleotides (A) G361 cells were treated in triplicate dishes for 60 min with A23187 (10 μM) or DMSO. Bacterially expressed AMPK (α1β2γ1 complex, inactive D157A mutant) was treated with increasing amounts of LKB1 (0.4–120 ng) and MgATP. Equivalent amounts of AMPK from G361 cell lysates and the bacterially expressed AMPK were then analyzed by SDS-PAGE and probed by western blotting with anti-pT172 and anti-α antibodies. (B) As in (A), except that HEK293 cells were treated with A23187 (10 μM), berberine (100 μM), or DMSO; in this experiment, the bacterially expressed heterotrimer was also incubated with increasing amounts of CaMKKβ (75–3,600 ng) and ATP, rather than LKB1. (C) G361 cells (left panel) or HEK293 cells (right panel) were treated for 60 min with or without A23187 (10 μM) or berberine (100 μM), and AMPK was immunoprecipitated with anti-α antibodies. AMPK activity (mean ± SD, n = 3) was then measured before and after subsequent phosphorylation using CaMKKβ and MgATP. Statistically significant from DMSO control (before CaMKK treatment) by one-way ANOVA with Dunnett’s multiple comparison test: ^∗^p < 0.05, ^∗∗∗^p < 0.001. (D) Model for the regulation of AMPK by adenine nucleotides, based on results in this paper.

First, we quantified the signal obtained by western blotting using anti-pT172 antibody in lysates of cells treated with and without A23187 and/or berberine. The signal was calibrated using equivalent amounts of bacterially expressed human α1β2γ1 complex (inactive α1-D157A mutant) phosphorylated by increasing amounts of LKB1 until phosphorylation reached a maximum. By making the assumption that this corresponded to stoichiometric phosphorylation, and quantifying the blots by densitometry, we were able to estimate the stoichiometry of phosphorylation in the intact cells. Figure 7A shows results from one experiment with three replicate dishes of control and A23187-treated G361 cells. This experiment was repeated five times, and the average extent of phosphorylation estimated in control cells was 4% ± 1%, increasing by an average of 3.6-fold to 13% ± 2% after A23187 treatment (mean ± SEM, n = 5, p = 0.0033 by paired t test). We used the same method to estimate the changes in Thr172 phosphorylation when HEK293 cells were treated with A23187 or berberine (Figure 7B). The estimated basal phosphorylation (27% ± 1%) was much higher in HEK293 than in G361 cells, consistent with the presence of LKB1. This increased to 36% ± 2% in response to A23187 and 50% ± 3% in response to berberine. A23187 and berberine thus caused 1.3-fold and 1.9-fold increases in Thr172 phosphorylation, respectively.

As a second approach, we immunoprecipitated AMPK from the cells and incubated the precipitates with recombinant CaMKKβ and MgATP to fully activate them. The treatment with A23187 in the G361 cells caused a 5.3-fold increase in AMPK activity, but subsequent incubation of the immunoprecipitates with CaMKKβ caused further large activations to reach similar final values (Figure 7C, left panel). This yielded estimates that A23187 caused a 5-fold increase in Thr172 phosphorylation, from 3.5% ± 0.7 to 18% ± 2%. A23187 and berberine both caused increases in AMPK activity in HEK293 cells, but subsequent incubation of the immunoprecipitates with CaMKKβ caused further large activations up to similar final values (Figure 7C, right panel). This yielded estimates that A23187 and berberine caused 1.6-fold and 2.6-fold increases in Thr172 phosphorylation, from 23% ± 3% to 37% ± 2% and 60% ± 4% respectively.

Thus, these two approaches were in reasonable agreement. In the LKB1 null G361 cells, Thr172 phosphorylation was initially very low (4%), increasing after A23187 treatment to 13%–18%. In HEK293 cells, which express LKB1, the basal phosphorylation was initially much higher, i.e., 23%–27%. This increased to 36%–37% in response to A23187 and 50%–60% in response to berberine. The increases in phosphorylation in G361 cells due to A23187 were therefore 4-fold, and the increases in HEK293 cells due to A23187 and berberine were 1.5- and 2-fold, respectively. Thus, although 50%–60% of the α subunit became phosphorylated in berberine-treated HEK293 cells, the actual increases in Thr172 phosphorylation were quite modest.

## Discussion

Three major conclusions, summarized in the model shown in Figure 7D, can be drawn from this study:

(1) Dephosphorylation of Thr172 by PP2Cα is inhibited by ADP and AMP, with the effects of both being antagonized by ATP. However, AMP is about 10-fold more potent than ADP.
(2) AMP promotes Thr172 phosphorylation in cell-free assays by LKB1, but not CaMKKβ; ADP had no effect with either upstream kinase. This effect of AMP was not due to contamination with a PPM family protein phosphatase (Sanders et al., 2007) because: (i) there was no Thr172 dephosphorylation in incubations with LKB1 and Mg^2+^ when ATP was omitted; (ii) the effect on LKB1-mediated phosphorylation and activation was specific to AMP; if it had been due to inhibition of dephosphorylation, both AMP and ADP should have been effective.
(3) In cell-free assays, a large (13-fold) allosteric activation of AMPK by AMP occurred even when the concentration of ATP was 5 mM, within the normal physiological range (Imamura et al., 2009). We also studied phosphorylation of the downstream target acetyl-CoA carboxylase in LKB1 null G361 cells, and in AMPK knockout embryo fibroblasts expressing a T172D mutant, treated either with berberine (which increases AMP) or A23187 (which increases Ca^2+^ and activates CaMKKβ). These experiments revealed that the allosteric effect of AMP in intact cells could be at least as important, if not more important, than the effect of increased Thr172 phosphorylation.

Our results confirm previous results showing that the effect of AMP on Thr172 dephosphorylation is mimicked by ADP (Xiao et al., 2011). By carrying out assays in the presence of 5′-nucleotidase, we also showed that the effect of ADP could only be partially explained by its breakdown to AMP during assays. However, we disagree with Xiao et al. (2011) in one respect. Xiao et al. (2011) stated that “ADP provides protection of AMPK from dephosphorylation across a similar range of concentrations as AMP,” whereas Figure 1 clearly shows that AMP is about 10-fold more potent than ADP, irrespective of the presence or absence of 5 mM ATP. Xiao et al. (2011) also stated that “the dose-response curve for AMP/ADP-mediated protection against (de)phosphorylation correlates with the binding curves for these nucleotides at the weaker, rather than the stronger, of the two binding sites.” However, reanalysis of their Figure 1B suggests that the half-maximal effect of AMP on dephosphorylation that they observed was at ≈7 μM. This is close to our estimate of 3 μM and much closer to their estimate of the K_D_ for AMP binding at the “tight” (2.5 μM), rather than the “weak” (80 μM), site. Our very similar estimates of EC_50_ for the effects of AMP on dephosphorylation (3 μM) and allosteric activation (5 μM, measured with 200 μM ATP) also throw doubt on the proposal by Xiao et al. (2011) that allosteric activation is due to binding at the tight site, whereas the effect on dephosphorylation is due to binding at the weak site. By contrast, our estimate of the EC_50_ for the effect of AMP to promote phosphorylation by LKB1 (160 μM, measured with 200 μM ATP) is closer to their estimate of the affinity for AMP at the weak site.

Unlike Oakhill et al. (2011), we have been unable to show that AMP promotes Thr172 phosphorylation by CaMKKβ, either previously (Hawley et al., 2005) or in this study, and now also find that ADP is unable to promote Thr172 phosphorylation by LKB1; the reasons for these discrepancies remain unclear.

Our results reinforce the view that AMP, not ADP, is the critical physiological activator of AMPK, and that the name AMP-activated protein kinase remains appropriate. We have confirmed that AMP acts via three mechanisms (Figure 7C), two of which (allosteric activation and promotion of Thr172 phosphorylation by LKB1) are brought about only by AMP and not ADP. The third (inhibition of Thr172 dephosphorylation) can also be caused by ADP, but AMP is about 10-fold more potent than ADP. Since AMP concentrations are usually about 10-fold lower than concentrations of ADP in intact cells (Hardie et al., 2011; Oakhill et al., 2012), changes in both nucleotides might contribute to the enhanced net Thr172 phosphorylation observed in cells subject to energy stress. However, inspection of Figure 1A (where we have added dashed vertical lines to indicate the estimated changes in AMP and ADP concentrations induced by berberine in G361 cells), suggests that the changes in AMP would have a larger effect on Thr172 dephosphorylation than would the changes in ADP. AMP also increases in response to berberine in G361 cells over a range where large effects on phosphorylation by LKB1 (Figure 2B) and on allosteric activation (Figure 3A) were observed in cell-free assays.

Some recent reviews (Carling et al., 2012; Oakhill et al., 2012) have cast doubt on the idea that AMP is the critical physiological regulator of AMPK. There appear to be two main arguments underlying this. First, allosteric activation is usually reported to be quite modest (often <2-fold), yet stoichiometric phosphorylation of Thr172 can produce >100-fold activation. However, we could demonstrate 13-fold allosteric activation by AMP (Figure 3A), even using an ATP concentration (5 mM) within the physiological range for unstressed cells (Imamura et al., 2009). In addition, the results in Figures 7A–7C show that the 4-fold increase in response to A23187 in G361 cells represents a change from only ≈4% to ≈16% of maximal, stoichiometric phosphorylation, whereas in HEK293 cells, where the basal phosphorylation was much higher (25%), the increases in response to A23187 and berberine were only 1.5-fold and 2-fold. Thus, although it is possible to obtain >100-fold activation in cell-free assays, the changes in Thr172 phosphorylation in intact cells occur over a much narrower range. Our experiments in G361 cells also suggest that allosteric activation of AMPK can be as important as the effect of Thr172 phosphorylation in the overall activation mechanism. Due to the lack of LKB1 in these cells, berberine (which increases AMP and ADP) and A769662 (which binds directly to AMPK) had no effect on Thr172 phosphorylation or on AMPK activity measured in washed immunoprecipitates. Despite this, both increased the phosphorylation of ACC, a well-established marker for AMPK activation in intact cells, suggesting that they were acting in these cells purely via allosteric activation (berberine had a larger effect on ACC phosphorylation than did A23187, which acts via increased Thr172 phosphorylation). These conclusions depend on the assumption that AMPK is the only kinase phosphorylating ACC at Ser79 in these cells, as already demonstrated using AMPK knockouts in mouse embryo fibroblasts, hepatocytes, and T cells (Foretz et al., 2010; Laderoute et al., 2006; Rolf et al., 2013). To test this, we made cells stably expressing a dominant-negative AMPK-α2 mutant and observed that the A23187-induced increase in AMPK activity was reduced by an amount (≈70%) similar to that of the increase in ACC phosphorylation; ACC phosphorylation induced by A769662 was similarly decreased (Figures 6A and 6B). While we cannot rule out the possibility that some of the residual ACC phosphorylation in G361 cells expressing the dominant-negative mutant was due to a kinase other than AMPK, this kinase would have to be activated by both A23187 and A769662, which seems inherently unlikely.

Our results in the AMPK knockout MEFs expressing the T172D mutant of AMPK-α1 (which retains allosteric activation by AMP; Stein et al., 2000) also confirm that allosteric activation is an important component of the overall activation mechanism. The T172D mutant cannot be phosphorylated at Thr172, yet berberine still enhanced the phosphorylation of ACC (Figure 6C). Moreover, in the AMPK knockout MEFs expressing wild-type α1, the effect of berberine on ACC phosphorylation was larger than that of A23187, whereas the reverse was true for the effects on AMPK activity. We propose that this is because the effect of berberine on ACC phosphorylation reflects a combination of phosphorylation and allosteric activation of AMPK induced by AMP, whereas the effect of A23187 is only due to enhanced phosphorylation. Our results with A769662 (Figure 4B), which causes allosteric activation and promotion of net Thr172 phosphorylation like AMP, despite binding at a different site (Göransson et al., 2007; Hawley et al., 2012), suggest that allosteric activation by A769662 is also at least as important as its effects on Thr172 phosphorylation.

It has been reported that treatment of LKB1^−/−^ MEFs with H_2_O_2_ or AICA ribonucleotide (AICAR) led to increased ACC phosphorylation (Shaw et al., 2004), similar to that shown in our results using berberine in the LKB1 null G361 cell line. Shaw et al. (2004) stated that “there was still a small, but reproducible, amount of ACC phosphorylation in response to these stimuli in LKB1-null MEFs.” By contrast, in LKB1 null mouse muscle (Göransson et al., 2007) or mouse hepatocytes (Foretz et al., 2010), there was no detectable ACC phosphorylation. The simplest explanation for these results is that the expression of CaMKKβ is higher in immortalized cells than in LKB1 null muscle or hepatocytes, so AMPK is essentially inactive in the latter and cannot be allosterically activated by AMP.

The second argument against the role of AMP as a physiological regulator (Carling et al., 2012; Oakhill et al., 2012) is that the affinities of the two exchangeable sites (sites 1 and 3) within the γ1 subunit were estimated to be similar for AMP, ADP, and free ATP^4−^ (Xiao et al., 2011). Since AMP is normally present at much lower cellular concentrations, it has been argued that it would be unable to compete with ADP or ATP at either regulatory site. However, we observed 13-fold allosteric activation by AMP even when conducting assays at 5 mM ATP. Under these conditions, allosteric activation by AMP occurred from 50–500 μM, corresponding nicely with our estimated increase (40–270 μM, see Figure 3A) in AMP in G361 cells treated with berberine. Thus, AMP can compete with ATP at the allosteric binding site(s), even when its concentration is one to two orders of magnitude lower than that of ATP. It is worth noting that the affinities for AMP, ADP, and ATP of sites 1 and 3 on the γ1 subunit were estimated indirectly by competition with fluorescent derivatives of ATP and ADP and by making assumptions that there were only two exchangeable sites for each nucleotide that did not interact (Xiao et al., 2011). However, results obtained with mutations of aspartate residues at all three sites (1, 3, 4) suggest that these sites do interact (Oakhill et al., 2010). Moreover, another structural analysis, in which the heterotrimer core was crystallized independently with AMP or ATP, suggested that the so-called nonexchangeable site 4 can bind ATP as well as AMP and that binding of ATP at site 4 would prevent binding of other nucleotides at site 3 (Chen et al., 2012). Thus, the assumptions used to estimate the affinities for nucleotides at sites 1 and 3 (Xiao et al., 2011) may not have been completely valid.

Another important conclusion from our results is that simply estimating Thr172 phosphorylation as a marker for AMPK activation, which is common in the literature, can yield misleading results in that it completely ignores the effects of allosteric activation. We would suggest that it is advisable to also monitor the phosphorylation of at least one validated downstream target for AMPK, such as ACC. Finally, our results with G361 cells show that AMPK can still phosphorylate downstream targets such as ACC in response to energetic stress, even in LKB1 null tumor cells, although the effects may be smaller than those in LKB1-expressing cells in that they would rely entirely on allosteric activation, without any changes in Thr172 phosphorylation.

## Experimental Procedures

### Effect of AMP and ADP on Thr172 Dephosphorylation

Rat liver AMPK (100 units/ml, 62.5 μg/ml) was incubated at 30°C in Na HEPES (50 mM; pH 7.0), NaCl (150 mM), Brij 35 (0.02% v/v), dithiothreitol (1 mM) with MgCl_2_ (5 mM), and sufficient recombinant PP2Cα to yield about 70% inactivation in the absence of added nucleotide. AMP, ADP, or ATP were added at concentrations indicated in the figure legends, and aliquots were removed for western blotting or kinase assays. Kinase assays were performed immediately with a further dilution of 100-fold, which was sufficient to prevent dephosphorylation during the assay. Where added, the 5′-ectonucleotidase CD73 was at 3.9 μg/ml (58 units/ml, where 1 unit is 1 nmol of AMP hydrolyzed per min); it was diluted 150-fold into final kinase assays, where it did not cause a significant effect.

### Effects of AMP and ADP on Thr172 Phosphorylation

Rat liver AMPK (20 units/ml, 12.5 μg/ml) was incubated in Na HEPES (50 mM; pH 7.0), NaCl (50 mM), Brij 35 (0.02% v/v), and dithiothreitol (1 mM) for 15 min at 30°C with sufficient PP2A_C_ to give ≈95% inactivation. Okadaic acid was added (5 μM) to inhibit PP2A_C_. Dephosphorylated AMPK (2 μg/ml) was then incubated with recombinant LKB1-STRAD-MO25 complex (4 μg/ml) or CaMKKβ (1.2 μg/ml) together with MgCl_2_ (5 mM) and ATP (200 μM) in the same buffer, and incubation continued for 10 min, with AMP or ADP at concentrations given in the figures or legends. Aliquots were removed at the times stated for kinase assays or western blotting. When added, 5′-nucleotidase was at 1.8 μg/ml; it was diluted a further 150-fold into final kinase assays and had no effect on these. To confirm that AMPK was not contaminated with a PPM phosphatase, we incubated AMPK that had not been treated with PP2A_C_ in buffer A with or without LKB1, MgCl_2_ (5 mM), or PP2Cα exactly as for the rephosphorylation experiments, but omitting ATP. After 15 min at 30°C, aliquots were subject to kinase assay or western blotting.

### AMPK Purification and Assay

Rat liver AMPK was purified as described previously (Hawley et al., 1996), except that the final size-exclusion chromatography was not on Sephacryl S-200, but on a Superdex 200 (HiLoad 16/60) column, equilibrated in buffer lacking fluoride and pyrophosphate. AMPK assays in solution were performed as previously described (Hardie et al., 2000), except that in some assays (specified in the figure legends) the concentration of ATP was increased from 0.2 to 1 or 5 mM, with MgCl_2_ concentrations also increased (5, 5.8, or 9.8 mM, respectively) to maintain a constant excess of [Mg^2+^] over [ATP]. Where allosteric activation by AMP was being studied, we used the *SAMS* peptide as substrate. For immunoprecipitate assays, AMPK was precipitated from lysates of G361 cells (50 μg protein) by incubation at 4°C for 2 hr on a roller mixer, with equal amounts of AMPK-α1 and -α2 antibodies noncovalently coupled to protein G Sepharose (GE Healthcare). After washing, immunoprecipitates were assayed for AMPK activity using the *AMARA* peptide (Hardie et al., 2000). One unit of AMPK activity phosphorylates 1 nmol of substrate per min at 30°C.

### Generation of G361 Cells Expressing a Dominant-Negative α2 Mutant

G361 cells stably expressing an integrated Flp recombinase target (FRT) site were generated using the Flp-In System (Invitrogen) following manufacturer’s instructions. Briefly, G361 cells were transfected with the pFRT*lac*Zeo plasmid using FuGENE (Promega) and washed into medium containing zeocin (100 μg/ml) after 48 hr. The medium was replaced every 3–4 days until foci of cells could be identified and expanded. The incorporation of the FRT site was confirmed by monitoring β-galactosidase activity. A construct encoding human AMPK-α2 (D157A mutant) was amplified using primers designed to encode a 5′-Kpn1 site and a C-terminal FLAG tag followed by a 3′-Xho1 site. The resulting PCR product was inserted into pcDNA5/FRT. The G361 cells containing an FRT site were transfected with Effectene (QIAGEN) in the absence of zeocin, using the plasmids pOG44 (encoding *Flp* recombinase) and pcDNA5/FRT/α2D157A at a ratio of 9:1. After 48 hr, hygromycin B (100 μg/ml) was added to the medium, and the medium was changed every 3–4 days until foci of cells could be identified and expanded.

### Estimation of Degree of Thr172 Phosphorylation in Intact Cells

A polycistronic plasmid expressing human α1β2γ1 was generated by the Division of Signal Transduction Therapy, University of Dundee, using previously described methodology (Neumann et al., 2003). This was used as a template to generate a plasmid encoding a kinase-inactive D157A mutant using the QuikChange Site-Directed Mutagenesis Kit (Stratagene). The plasmid was expressed in bacteria and purified using a HisTrap column (GE Healthcare) as described previously (Hawley et al., 2012). The protein was incubated with increasing amounts of LKB1-STRAD-MO25 complex (or CaMKKβ, where specified), ATP (200 μM), and MgCl_2_ (5 mM) for 15 min at 30°C in 50 mM Na HEPES (pH 7.4), 1 mM dithiothreitol, 0.02% (v/v) Brij 35. The reaction was stopped by the addition of SDS, with further incubation at 70°C for 10 min. Samples (corresponding to 20–40 ng of AMPK) were loaded onto the same Bis-Tris 4%–12% polyacrylamide gels as lysates (20–40 μg protein) from cells that had been treated with DMSO, A23187, or berberine. Membranes were probed with the indicated antibodies, and band intensities were quantified using LI-COR Odyssey software.

In a second approach, cell lysates were immunoprecipitated with AMPK-α1 and AMPK-α2 antibodies covalently bound to protein G Sepharose. The Sepharose beads were incubated with CaMKKβ and MgATP for 30 min at 30°C and washed several times in Na HEPES buffer (pH 7.4) to remove CaMKKβ. AMPK activity was then determined using the *AMARA* peptide as above.

### Estimation of Cellular Adenine Nucleotide Ratios and Concentrations

Estimation of cellular ADP:ATP ratios were performed by capillary electrophoresis of perchloric acid extracts as described previously (Hawley et al., 2010), except that peaks were detected by absorbance at 254 nm. AMP:ATP ratios were estimated from ADP:ATP by assuming that the adenylate kinase reaction was at equilibrium, so that AMP:ATP = K_eq_.(ADP:ATP)^2^ (Hardie and Hawley, 2001), where K_eq_ = 1.05 (Lawson and Veech, 1979).Total ATP concentrations ([ATP]) were estimated from the ADP:ATP ratios by assuming that [ATP] + [ADP] + [AMP] was 5 mM, so that [ATP] = 5/(1 + ADP:ATP + AMP:ATP). Estimates for [AMP] and [ADP] could then be calculated from the values for [ATP], ADP:ATP, and AMP:ATP obtained.

### Presentation of Data and Statistical Analysis

Unless stated otherwise, data presented are mean ± SEM (n = 3), with all replicates in intact cell experiments from separate dishes of cells. Unless stated otherwise, statistical analysis (GraphPad Prism 5 for Mac OSX) was by ANOVA, using Bonferroni’s multiple comparison test of selected data sets (^∗^p < 0.05, ^∗∗^p < 0.01, ^∗∗∗^p < 0.001; ns, not significant).

### Additional Experimental Procedures

Additional experimental procedures are provided in the Supplemental Information.

## Supplemental Information

Document S1. Supplemental Experimental Procedures and Table S1
