# Supplementary material for: AMP Is a True Physiological Regulator of AMP-Activated Protein Kinase by Both Allosteric Activation and Enhancing Net Phosphorylation
Source: Cell Metab. 2013 Oct 1;18(4):556–66. doi: 10.1016/j.cmet.2013.08.019 (PMC3791399; doi:10.1016/j.cmet.2013.08.019)
Supplement: Document S1. Supplemental Experimental Procedures and Table S1 [file mmc1.pdf]

Cell Metabolism, Volume *18*

Supplemental Information

AMP Is a True Physiological Regulator of AMP-Activated Protein Kinase by Both Allosteric Activation and Enhancing Net Phosphorylation

Graeme J. Gowans, Simon A. Hawley, Fiona A. Ross, and D. Grahame Hardie

Supplementary Data

Table S1: Estimates for adenine nucleotide ratios and concentrations in G361 cells treated with different AMPK activators (related to Fig. 5). Values were estimated as described in Supplementary Experimental Procedures, and are mean ± SEM (n = 3). **P<0.01, ***P<0.001, significantly different from control by 1 way ANOVA, using Dunnett's multiple comparison test compared with control. Values for A23187 and A769662 were not significantly different from control.

	Control	A23187	Berberine	A769662
ADP:ATP	0.094 ± 0.004	0.113 ± 0.004	0.262 ± 0.029***	0.109 ± 0,006
AMP:ATP	0.0093 ± 0.0008	0.0135 ± 0.0010	0.0738 ± 0.0166**	0.0125 ± 0.0014
ATP (mM)	4.53 ± 0.02	4.44 ± 0.02	3.75 ± 0.12***	4.46 ± 0.03
ADP (µM)	426 ± 16	503 ± 16	975 ± 72***	484 ± 24
AMP (µM)	42.3 ± 3.4	60.0 ± 4.0	273 ± 51***	55.7 ± 5.6

Supplementary Experimental Procedures

Materials

Berberine chloride and A23187 were from Sigma. A769662 was synthesized as described previously (Iyengar et al., 2005). STO609 was from Tocris Bioscience and zeocin and hygromycin B from Invitrogen. The anti-pT172 antibody was from Cell Signalling Technology and antibodies against actin and the FLAG epitope from Sigma. Affinity purified antibodies against ACC (pS79) (Hawley et al., 2003) and AMPK-α1 and -α2 (Woods et al., 1996) were described previously; the "anti-AMPK-α" antibody is an equal mixture of anti-α1 and -α2 antibodies. Secondary antibodies were from Li-Cor Biosciences. The 5'-nucleotidase was recombinant human CD73 from R&D Systems (Cat. # 5795-EN-010). Human CaMKKβ was expressed as a GST fusion and purified from *Escherichia coli* (Hawley et al., 2005). Human LKB1-STRAD-MO25 complex was expressed in an insect cell baculovirus system (Jaleel et al., 2006). PP2Cα was expressed and purified from *E. coli* (Davies et al., 1995), and $PP2A_C$ purified from bovine heart (Cohen et al., 1988). The GST fusion of rat AMPK-α1 (1-310) was expressed and purified from *E. coli* (Goransson et al., 2007). DNA encoding human AMPK-α2 (1-310) was amplified by PCR using the primers:
5'-CGCGGATCCGCGATGGCTGAGAAGCAGAAGCAC-3' and
5'-CCGCTCGAGCGGTCAACTGTTCATTACTTCTGATTC-3' and inserted into the vector pGEX6P2. The plasmid was used to transform *E. coli* strain BL21(DE3), which was grown in LB medium until absorbance at 600 nm reached 0.6, when isopropyl β-D-1-thiogalactopyranoside (1 mM) was added to induce protein expression. Bacterial cells were harvested by centrifugation, lysed using a pestle and mortar and resuspended in 50 mM Tris/HCl pH 8.5, 500 mM NaCl, 1 mM dithiothreitol, 1 mM EGTA, 1 mM EDTA with Complete Protease Inhibitor mix (Roche). The lysate was clarified by centrifugation, purified on glutathione-Sepharose (GE Healthcare) and eluted with 50 mM Na Hepes pH 8.0, 200 mM NaCl, 20 mM glutathione. The purified GST-kinase domain fusions were incubated with LKB1-STRAD-MO25 complex for 30 min at 30°C to allow maximal Thr172 phosphorylation, and were then assayed for AMPK activity using the *AMARA* peptide, as described below.

Cell culture and lysis

G361 cells were from the European Collection of Cell Cultures. The cells were grown in McCoy's 5A medium containing 10% (v/v) fetal bovine serum (FBS), 100 IU/ml penicillin and 100 µg/ml streptomycin. AMPK-$\alpha1^{-/-}$ -$\alpha2^{-/-}$ mouse embryo fibroblasts (MEFs), a gift from Benoit Viollet, were grown as described previously (Laderoute et al., 2006). They were transfected with DNAs encoding myc-tagged AMPK-α1, β2 and FLAG-tagged γ1, all human sequences. The myc-α1 either had the wild type sequence or a T172D mutation, made using the Stratagene Quikchange II Site-directed mutagenesis system. MEFs were grown to 90% confluency in 6 cm dishes and were transfected using Lipofectamine 2000 (Invitrogen). Cells were washed into Opti-MEM just prior to transfection, and then back into standard growth medium after 5 hr. After 48 hr, they were treated with A23187 (10 µM), berberine (300 µM) or an equivalent quantity of DMSO for 1 hr prior to preparation of cell lysates for analysis.

After various treatments as indicated in Figure legends, cells were rinsed rapidly with ice-cold phosphate-buffered saline to remove the medium and then lysed in situ using 0.3 ml of ice-cold lysis buffer (50 mM Tris/HCl pH 7.2, 1 mM EGTA, 1 mM EDTA, 50 mM NaF, 1 mM Na pyrophosphate, 1% (w/v) Triton X-100, 0.1 mM phenylmethane sulphonyl fluoride, 1 mM dithiothreitol, 0.1 mM benzamidine, and 5 mg/ml soybean trypsin inhibitor). The lysates were centrifuged (4°C, 10 min, 21,000 x g) and the supernatants frozen for later analysis.

SDS PAGE and Western blotting

For analysis of ACC phosphorylation this was performed using precast Novex NuPAGE Tris-Acetate 3%-8% gradient polyacrylamide gels in the Tris-Acetate SDS buffer system, or for analysis of all other proteins using precast Novex NuPAGE Bis-Tris 4%-12% gradient polyacrylamide gels in the MOPS buffer system (Invitrogen). Proteins were transferred to nitrocellulose membranes (Bio-Rad) using the Xcell II Blot Module (Invitrogen). Membranes were blocked in Li-Cor Odyssey blocking buffer for 1 hr (Li-Cor Biosciences), probed with the appropriate antibody in Odyssey blocking buffer, and detection performed using the appropriate secondary antibody coupled to IR680 or IR800 dye. Membranes were scanned using the Li-Cor Odyssey IR imager.

Supplementary references

Cohen, P., Alemany, S., Hemmings, B.A., Resink, T.J., Strålfors, P., and Lim, T.H. (1988). Protein phosphatase-1 and protein phosphatase-2A from rabbit skeletal muscle. Methods Enzymol. *159*, 390-408.

Davies, S.P., Helps, N.R., Cohen, P.T.W., and Hardie, D.G. (1995). 5'-AMP inhibits dephosphorylation, as well as promoting phosphorylation, of the AMP-activated protein kinase. Studies using bacterially expressed human protein phosphatase-2Cα and native bovine protein phosphatase-2A_C. FEBS Lett. *377*, 421-425.

Goransson, O., McBride, A., Hawley, S.A., Ross, F.A., Shpiro, N., Foretz, M., Viollet, B., Hardie, D.G., and Sakamoto, K. (2007). Mechanism of action of A-769662, a valuable tool for activation of AMP-activated protein kinase. J. Biol. Chem. *282*, 32549-32560.

Hawley, S.A., Boudeau, J., Reid, J.L., Mustard, K.J., Udd, L., Makela, T.P., Alessi, D.R., and Hardie, D.G. (2003). Complexes between the LKB1 tumor suppressor, STRADα/β and MO25α/β are upstream kinases in the AMP-activated protein kinase cascade. J. Biol. *2*, 28.

Hawley, S.A., Pan, D.A., Mustard, K.J., Ross, L., Bain, J., Edelman, A.M., Frenguelli, B.G., and Hardie, D.G. (2005). Calmodulin-dependent protein kinase kinase-beta is an alternative upstream kinase for AMP-activated protein kinase. Cell Metab. *2*, 9-19.

Iyengar, R.R., Judd, A.S., Zhao, G., Kym, P.R., Sham, H.L., Gu, Y., Liu, G., Zhao, H., Clark, R.E., Frevert, E.U., et al. (2005). Thienopyridines as AMPK activators for the treatment of diabetes and obesity. Patent No. US 2005/0038068 A1.

Jaleel, M., Villa, F., Deak, M., Toth, R., Prescott, A.R., Van Aalten, D.M., and Alessi, D.R. (2006). The ubiquitin-associated domain of AMPK-related kinases regulates conformation and LKB1-mediated phosphorylation and activation. Biochem. J. *394*, 545-555.

Laderoute, K.R., Amin, K., Calaoagan, J.M., Knapp, M., Le, T., Orduna, J., Foretz, M., and Viollet, B. (2006). 5'-AMP-activated protein kinase (AMPK) is induced by low-oxygen and glucose deprivation conditions found in solid-tumor microenvironments. Mol. Cell. Biol. *26*, 5336-5347.

Woods, A., Salt, I., Scott, J., Hardie, D.G., and Carling, D. (1996). The α1 and α2 isoforms of the AMP-activated protein kinase have similar activities in rat liver but exhibit differences in substrate specificity *in vitro*. FEBS Lett. *397*, 347-351.
